# Encapsulation of cathode in lithium-sulfur batteries with a novel two-dimensional carbon allotrope: DHP-graphene

**DOI:** 10.1038/s41598-017-15010-7

**Published:** 2017-11-02

**Authors:** Yingxiang Cai, Yuqing Guo, Bo Jiang, Yanan Lv

**Affiliations:** Department of Physics, School of Science, Nanchang University, Nanchang, 330031 China

## Abstract

Sulfur cathodes in lithium-sulfur (Li-S) batteries still suffer from their low electronic conductivity, undesired dissolution of lithium polysulfide (Li_2_S_*n*_, 3 ≤ *n* ≤ 8) species into the electrolyte, and large degree volume change during the cycle. To overcome these problems, an effective encapsulation for the sulfur cathode is necessary. By means of particle swarm optimization (PSO) and density functional theory (DFT), we have predicted a stable metallic two-dimensional *sp* ^2^-hybridized carbon allotrope (DHP-graphene). This carbon sheet can prevent S atoms from cathode entering electrolyte. However, Li-ions can shuttle freely due to the increasing difference in Li-ions concentration between electrolyte and cathode along with the potential difference between cathode and anode during charge-discharge cycles. In addition, versatile electronic band structures and linear dispersion are found in DHP-graphene nanoribbons but only metallic band structure occurs for DHP-graphene nanotubes.

## Introduction

The electrical storage with battery is one of the most significant requirements nowadays^1^. Li-ion rechargeable batteries have played a key role in commercial applications such as portable electronic devices and electric vehicles due to their superior energy density^1–6^. However, with growing energy demand for business, existing Li-ion rechargeable batteries have approached their bottlenecks and can not meet the critical demand for batteries energy density. Thus, a new high-energy storage system is urgently needed^7–9^. Li-S rechargeable batteries are widely considered to be the next-generation high-energy storage system, due to their high theoretical capacity (1675 mAh/g) and energy density (2600 Wh/kg)^10,11^. In addition, sulfur element is abundant and environmental-friendly for long-term use^12^. Despite their many advantages, Li-S batteries still exist some problems to be solved. Firstly, the low electronic conductivity of sulfur and its discharge products (Li_2_S and Li_2_S_2_) limits the actual utilization of active materials^10^. Secondly, polysulfides (Li_2_S_*n*_, 3 ≤ *n* ≤ 8), the by-products during the charge-discharge, can highly solubilize in the electrolyte, and freely shuttle between cathode and anode, which is called “shuttle phenomenon”. It will lead to low coulomb efficiency, loss of active materials and short cycle life^2,13^. Lastly, large degree expansion and shrinkage of cathode volume during the lithiation/delithiation result in the stability damage and capacity decay of cathode^10,14^.

In order to solve the above problems, a cathode material that not only has good electrical conductivity but also can immobilize the polysulfides is required. Various sulfur-based composites including polymer-sulfur composites^15–18^, carbon-sulfur composites^19–22^, and carbon-polymer-sulfur composites^23–25^, have been investigated. Among the available carbon-based materials, graphene inherently shows excellent electrical conductivity, mechanical flexibility and high specific surface area^26–29^. However, in the cathode of sulfur-graphene composites, graphene acts as a substrate loading sulfur failed to immobilize the polysulfides in the cathode to alleviate the shuttle phenomenon^5,30^. To overcome this drawback, both replacing the graphene with graphene oxide and encapsulating graphene-sulfur composites with polymers are effective approaches to alleviating shuttle phenomenon, but suffer from the electrical conductivity decreasing instead^30^. Moreover, some metal oxides such as Ti_4_O_7_^31,32^ and MnO_2_^33^ can alleviate the shuttle phenomenon by converting soluble polysulfides to insoluble low order sulfides, but their electronic conductivity is also much lower than carbon materials just like graphene oxide and polymers. Archer’s group has successfully alleviated the shuttle phenomenon and meanwhile enhanced cathode electrical conductivity to some extent by encapsulating sulfur with porous hollow carbon^34^. However, the effective encapsulation of sulfur cathode with high electrical conductivity is still an open problem urgent to be solved for the practical application of Li-S batteries.

Herein, we firstly predict a new two-dimensional (2D) *sp* ^2^-hybridized porous carbon allotrope named as DHP-graphene and evaluate its dynamic and thermodynamic stability. Secondly, The electronic band structures of DHP-graphene and its derivatives, including nanotubes and nanoribbons, are investigated. Finally, the potential application of DHP-graphene in the cathode encapsulation of Li-S is evaluated. The main purpose of this study is to demonstrate the feasibility of alleviating the shuttle phenomenon and maintaining high electrical conductivity at the same time by using this novel carbon allotrope as the cathode of Li-S batteries.

## Results and Discussion

**Figure 1:**
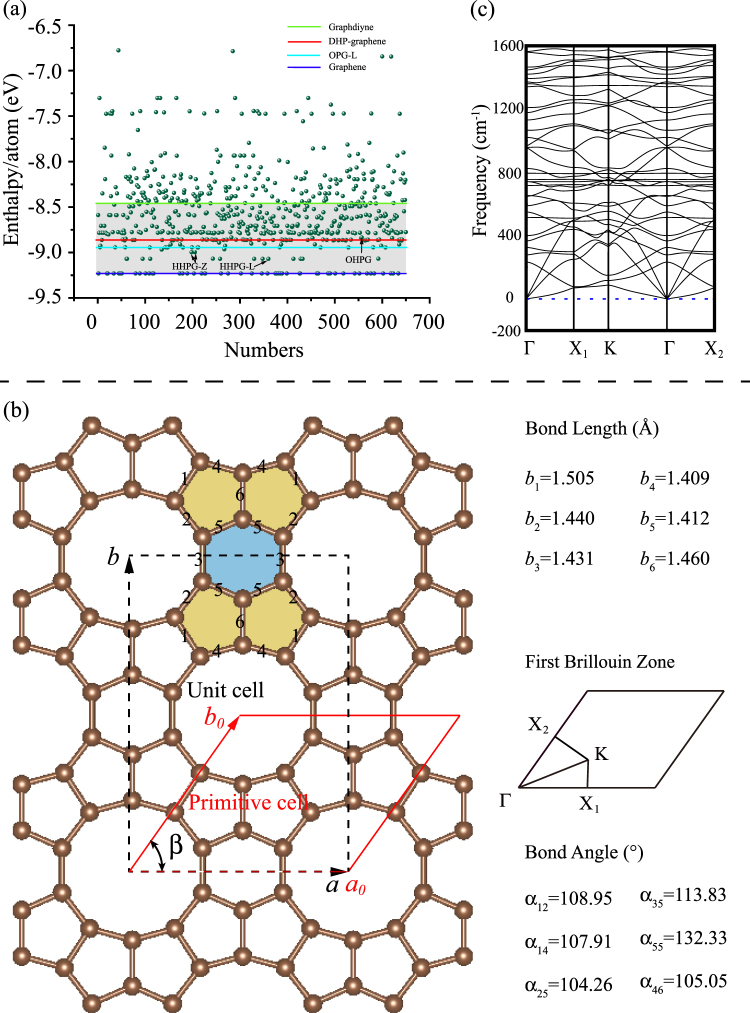
(**a**) Enthalpy evolution for a 12-atom carbon system during structure research. (**b**) Two-dimensional crystal structure of DHP-graphene and its bond lengths, bond angles and first Brillouin Zone. (**c**) Phonon dispersion along the typical directions.

The enthalpy evolution for a 12-atom carbon system is shown in Fig. 1(a). Among all generated 2D structures, graphene is the most stable. OPG-L^35^ predicted by Feng *et al*. is found again in this structure research. In addition, a few carbon allotropes, such as HHPG-L, HHPG-Z and OHPG, also have lower enthalpies but they are dynamic unstable (see the supporting information). Fortunately, a dynamic stable 2D *sp* ^2^ hybridized carbon allotrope with low enthalpy attracts our attentions. Its structure is shown in Fig. 1(b). This carbon sheet consists of **d**ecagonal, **h**exagonal and **p**entagonal rings and thus is named as DHP-graphene. Its unit cell is emphasized by black dashed lines, and the lattice constants *a* and *b* are 7.08 Å and 9.92 Å, respectively. DHP-graphene belongs to the 2D space group of Cmm. The primitive cell of DHP-graphene is highlighted by a red parallelogram. Six independent bond lengths of DHP-graphene are 1.505 Å, 1.440 Å, 1.431 Å, 1.409 Å, 1.412 Å, and 1.460 Å for *b*_1_, *b*_2_, *b*_3_, *b*_4_, *b*_5_, and *b*_6_, respectively. The average bond length (1.443 Å) of DHP-graphene is a little longer than that of graphene (1.42 Å). The bond angle is 108.95°, 107.91°, 104.26°, 113.83°, 132.33° and 105.05° for *α*_12_, *α*_14_, *α*_25_, *α*_35_, *α*_55_ and *α*_46_, respectively. The deviation of bond length and angle from ideal *sp* ^2^ carbon (graphene) results in the increasing of total energy. However, DHP-graphene is still more thermodynamic stable than graphdiyne which has been experimentally synthesized by Li *et al*.^36^ Similar to other carbon sheets, the DHP-graphene is also a flexible structure, and many potential applications need to be investigated. The first Brillouin zone is also shown in Fig. 1(b). The coordinates (*x*,*y*) of ***K*** point are calculated by the formulas of $y=({{b}_{0}}^{* }\,-\,{{a}_{0}}^{* }\,cos\,\beta )/2{{b}_{0}}^{* }\,{sin}^{2}\,\beta$ and $y=({{b}_{0}}^{* }\,-$ ${{a}_{0}}^{* }\,cos\,\beta )/2{{b}_{0}}^{* }\,{sin}^{2}\,\beta$, where the ${{a}_{0}}^{* }$ and ${{b}_{0}}^{* }$ are the basic vectors of reciprocal space, and the *β* is the angle between ${{a}_{0}}^{* }$ and ${{b}_{0}}^{* }$. Figure 1(c) shows the phonon dispersion along the high symmetry direction of DHP-graphene. Since no imaginary frequencies occur, the DHP-graphene is dynamic stable.

**Figure 2:**
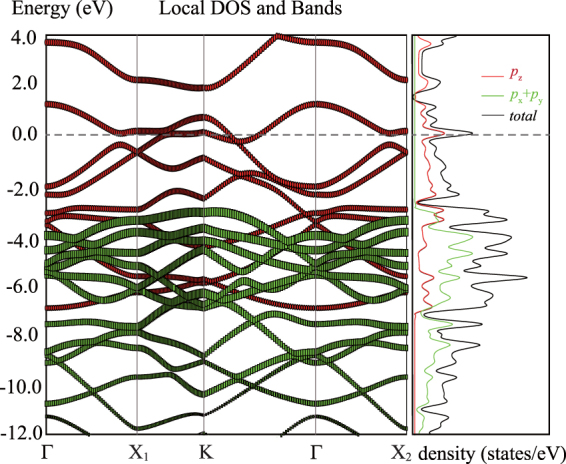
The energy band and density of states (DOS) for DHP-graphene.

For a material to be applied in the cathode of Li-S barriers, high electronic conductivity is necessary. Therefore, we investigate the electronic band structure and density of state (DOS) of DHP-graphene as shown in Fig. 2. It can be found that DHP-graphene is a metallic carbon allotrope as a distinct DOS peak occurs at the Fermi energy level (E_*f*_). The projected DOS shows that the DOS near E_*f*_ is mainly ascribed to the contribution of *p*_*z*_ orbital electron and slightly from *p*_*x*_ and *p*_*y*_ orbital electron. Thus the C-C bonding type is dominant *π* bonding below E_*f*_ and *π* ^*^ antibonding above E_*f*_. It implies high electronic conductivity of DHP-graphene. In contrast with graphene oxide, polymers or metal oxide, if the DHP-graphene is used as the cathode of Li-S batteries, the present disadvantage of low electronic conductivity could be improved significantly.

**Figure 3:**
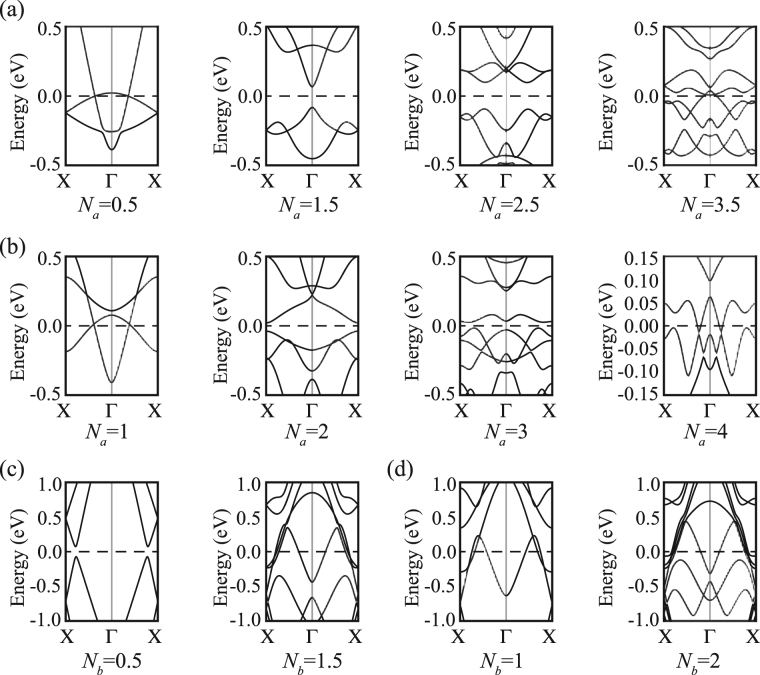
Energy bands for armchair-edged DHP-graphene nanoribbons with (**a**) half-integer *N*_*a*_ = 0.5, 1.5, 2.5, 3.5 and (**b**) integer *N*_*a*_ = 1, 2, 3, 4. Energy bands for zigzag-edged DHP-graphene nanoribbons with (**c**) half-integer *N*_*b*_ = 0.5, 1.5, and (**d**) integer *N*_*b*_ = 1, 2.

Similar to graphene or other 2D materials, DHP-graphene also has two kinds of derivatives, nanoribbons and nanotubes. In this study, zigzag and armchair DHP-graphene nanoribbons (DHP-GNRs) are obtained by tailoring the DHP-graphene along lattice vectors $\vec{a}$ and $\vec{b}$ as shown in Fig. 1(b). The edge carbon atoms are passivated by hydrogen atoms. The width of DHP-GNRs is characterized by *N*_*a*_ and *N*_*b*_, where the *N*_*a*_ and *N*_*b*_ are the ratios of the width of DHP-GNR to lattice constant of $\vec{a}$ and $\vec{b}$, respectively. When the *N*_*a*_ and *N*_*b*_ are integers, DHP-GNRs have inversion symmetry center (ISC). Otherwise, *N*_*a*_ and *N*_*b*_ are half-integers and no ISC exists. Strikingly, DHP-GNRs exhibit diversified electronic properties as shown in Fig. 2. Armchair DHP-GNR can be either metallic or semiconducting, which depends on the *N*_*a*_ (see Fig. 3(a) and (b)). When the *N*_*a*_ is 0.5, 1, 3.5 and 4, the DHP-GNRs are metallic. And the dispersion is almost linear for both valence band maximum (VBM) and conduction band minimum (CBM) near Fermi energy level. In contrast, the DHP-GNRs are direct bandgap semiconductors as *N*_*a*_ = 1.5, 2 and 2.5. And the bandgap is 150, 60, and 190 meV and occurs at Γ, X point and along Γ-X, respectively. When the *N*_*a*_ is 3, DHP-GNR is a semiconductor too, but has a 42 meV indirect bandgap. Figure 3(c) and (d) show the electronic band structure of zigzag DHP-GNRs. We find that all zigzag DHP-GNRs are metallic except for the one with *N*_*b*_ = 0.5. It is worth noting that the dispersion is linear for both VBM and CBM. Thus, the electron and hole carriers are massless, and their mobility are very high. In contrast with zero bandgap graphene, this DHP-GNR exhibits distinct advantages using for semiconductor devices including field-effect transistor due to its open bandgap and linear dispersion.

**Figure 4:**
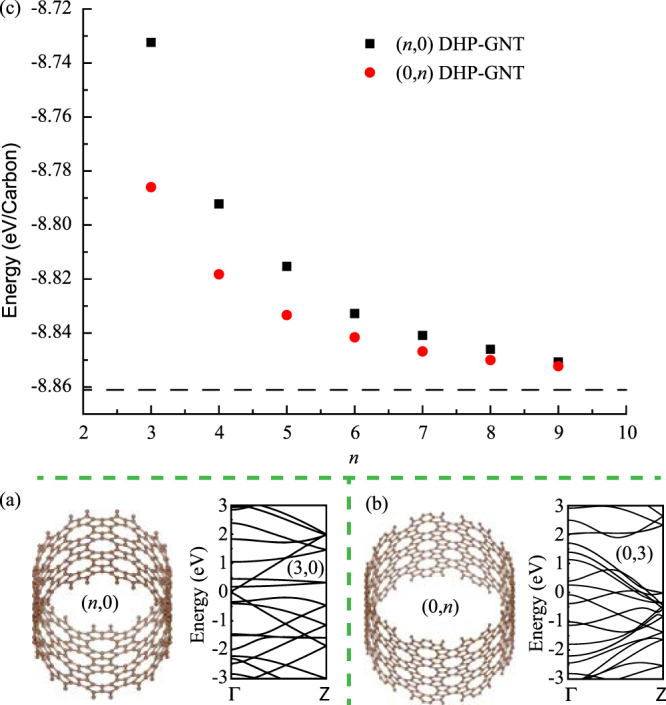
(**a**) The structure of zigzag (*n*, 0) DHP-GNT and the energy band structure for (3, 0) DHP-GNT. (**b**) The structure of armchair (0, *n*) DHP-GNT and the energy band structure for (0, 3) DHP-GNT. (**c**) The change of structural stability with the *n* used to characterize the size of DHP-GNTs.

The other kind of derivatives of DHP-graphene are DHP-graphene nanotubes (DHP-GNTs). By rolling DHP-graphene along vectors $\vec{a}$ and $\vec{b}$, (*n*, 0) zigzag DHP-GNTs and (0, *n*) armchair DHP-GNTs can be achieved. Two typical structures of DHP-GNTs are shown in Fig. 3(a) and (b), respectively. We also investigate the electronic band structure of DHP-GNTs and find that two smallest DHP-GNTs, (3, 0) and (0, 3) DHP-GNTs, are metallic. With the increasing of the diameter, the surface curvature of DHP-GNT will decrease. While the diameter of DHP-GNT tends to infinity, it can be viewed as DHP-graphene. Thus, it can be inferred that all DHP-GNTs are metallic. The width-dependent stability of DHP-GNTs is shown in Fig. 4(c). It can be seen that the energy per atom of DHP-GNTs decreases with the increasing of diameters, and finally approach the energy of DHP-graphene, i.e. −8.86 eV. In addition, the (0, *n*) DHP-GNT is more stable than the corresponding (*n*, 0) DHP-GNT.

**Figure 5:**
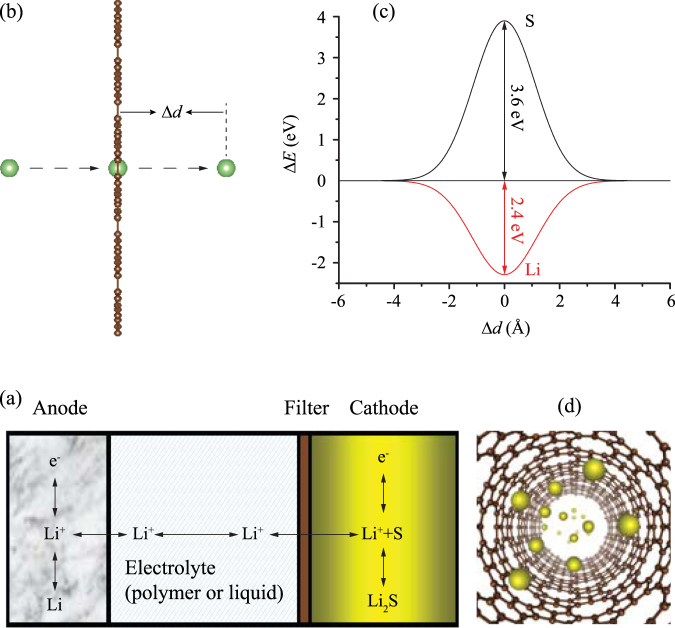
(**a**) DHP-graphene used as a filter to encapsulate the cathode of Li-S batteries. (**b**) Illustrating the process of one atom passing through decagonal rings. (**c**) The energy changes for Li and S atoms penetrating DHP-graphene. (**d**) Sulfur atoms are confined the inside of the DHP-GNTs.

The main issues faced in Li-S batteries including low coulomb efficiency, loss of active materials and short cycle life, are arisen from the shuttle phenomenon^37^. Therefore, alleviating the shuttle phenomenon is necessary for the commercial application of Li-S batteries. The natural decagonal rings provide the possibility for DHP-graphene used as a selective filter for Li, S and soluble polysulfides (Li_2_S_*n*_, 3 ≤ *n* ≤ 8) as illustrated in Fig. 5(a). Herein, we only investigate the process of a Li and a S passing through a decagon ring since it is impossible for soluble polysulfides and long chain S_*n*_^−2^ to penetrate through a decagon ring due to its small size (4.43–4.72 Å). A parameter, Δ*d*, is defined as the vertical distance between Li (or S) atom and DHP-graphene as shown in Fig. 5(b). The energy barrier for S passing through decagon ring is 3.6 eV at Δ*d* = 0 as shown in Fig. 5(c). Such a barrier is high enough to prevent S from cathode entering electrolyte. Therefore, the shuttle phenomenon could be effectively avoided. In contrast, no energy barriers for Li passing through the decagon ring of DHP-graphene. Surprisingly, the total energy decreases up to 2.4 eV when Li locates at the center of decagon ring. It implies the Li tends to adsorb on the DHP-graphene when it is used as a filter to encapsulate the cathode. During charge-discharge cycles, the increasing difference in Li-ions concentration between electrolyte and cathode combined with the potential difference between cathode and anode will enable Li-ions to pass through DHP-graphene. Likewise, metallic DHP-GNTs can also be used for the cathode of Li-S batteries. Unlike traditional sulfur-carbon nanotube based cathodes in which S atoms adsorb on the outside surfaces of CNTs^5^, S atoms will be confined into nanotubes for S and DHP-GNTs based cathode as illustrated in Fig. 5(d). But Li-ions can shuttle across DHP-GNTs through decagon rings. Since DHP-GNTs are metallic, the electronic conductivity will be improved significantly relative to traditional sulfur cathode. In addition, the large degree expansion and shrinkage of cathode volume during the lithiation/delithiation can be decreased due to DHP-GNTs’ intrinsic strength.

## Summary

In summary, we have proposed a new 2D porous carbon allotrope (DHP-graphene) based on particle swarm optimization and density functional theory. This 2D carbon structure is flexible and belongs to Cmm space group. By phonon dispersion calculations, DHP-graphene is proved to be dynamic stable. As far as thermodynamic stability is concerned, it is more stable than graphdiyne which is also a 2D carbon allotrope experimentally synthesized. Electronic band calculations show that both DHP-graphene and its derivative (DHP-GNTs) are metallic carbon allotropes. However, the other derivative, DHP-GNR can be metallic or semiconducting, which depends on the width of nanoribbons and edge structure. Versatile electronic structures make DHP-graphene and its derivatives have potentials to be used for all carbon-based nano devices. Our DFT calculations show DHP-graphene could be used as a filter to encapsulate the cathode of Li-S batteries due to its decagon carbon rings. A 3.6 eV energy barrier prevent S atoms entering electrolyte. However, Li-ions can shuttle across DHP-graphene with the help of Li-ions concentration and potential differences during charge-discharge cycles. In addition, if DHP-GNTs is used for the cathode of Li-S batteries, the volume expansion of cathode can be alleviated because S atoms are confined the inside of DHP-GNT but not adsorb its outside surface.

## Methods

Crystal structure predictions were performed using particle-swarm optimization (PSO) algorithm within the evolutionary scheme as implemented in the CALYPSO code^38^. The population size and the number of generation were set to be 26, and there were 12 carbon atoms in the crystal cell. Density functional theory (DFT) calculations were carried out using general gradient approximation (GGA)^39^ as implemented in the Vienna ab initio simulation package (VASP)^40,41^. The interactions between the nucleus and valence electrons of carbon were described by the projector augmented wave (PAW) method^42^. A plane-wave basis with a cutoff energy of 400 eV was used to expand the wave functions for all carbon structures investigated in this work. The geometries of carbon structures including the atomic position and lattice parameters were fully relaxed until the residual forces on atoms were less than 0.0001 eV·Å^−1^. The Brillouin Zone sample meshes were 5 × 5 × 1 for DHP-graphene, 1 × 1 × 5 for one-dimensional carbon nanotube and 1 × 1 × 5 for nanoribbons, in our calculations. The phonon band structure for this new two-dimensional carbon allotrope was calculated using the direct supercell method implemented in the Phonopy program^43^.

## Electronic supplementary material

Supporting Information
