# Supplementary material for: Encapsulation of cathode in lithium-sulfur batteries with a novel two-dimensional carbon allotrope: DHP-graphene
Source: Sci Rep. 2017 Nov 2;7:14948. doi: 10.1038/s41598-017-15010-7 (PMC5668302; doi:10.1038/s41598-017-15010-7)
Supplement: Supplementary file 1 — Supporting Information [file 41598_2017_15010_MOESM1_ESM.pdf]

Supporting Information

Encapsulation of cathode in lithium-sulfur batteries with a novel two-dimensional carbon allotrope: DHP-graphene

Yingxiang Cai[a*], Yuqing Guo[a], Bo Jiang[a], Yanan Lv[a]
[*]Corresponding Author, E-mail: yingxiangcai@ncu.edu.cn
[a] Department of Physics, Nanchang University, Jiangxi, Nanchang 330031, P.R. China.

The following three figures show the crystal structures and phonon dispersions of HHPG-L, HHPG-Z and OHPG. Distinct imaginary frequencies show their dynamic instabilities.

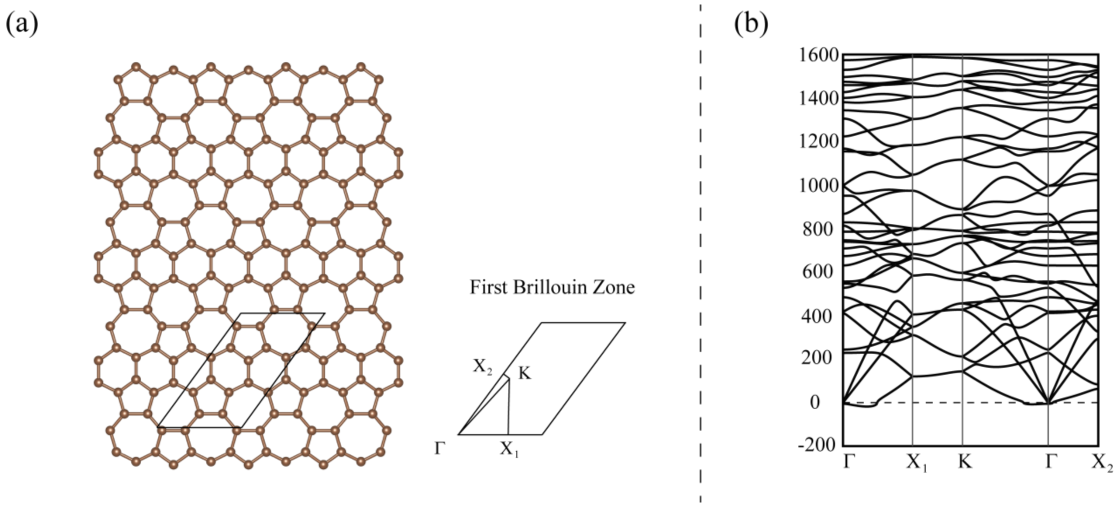

Fig. S1. (a) Two-dimensional crystal structure of HHPG-L and its first Brillouin Zone. (b) Phonon dispersion along the typical directions.

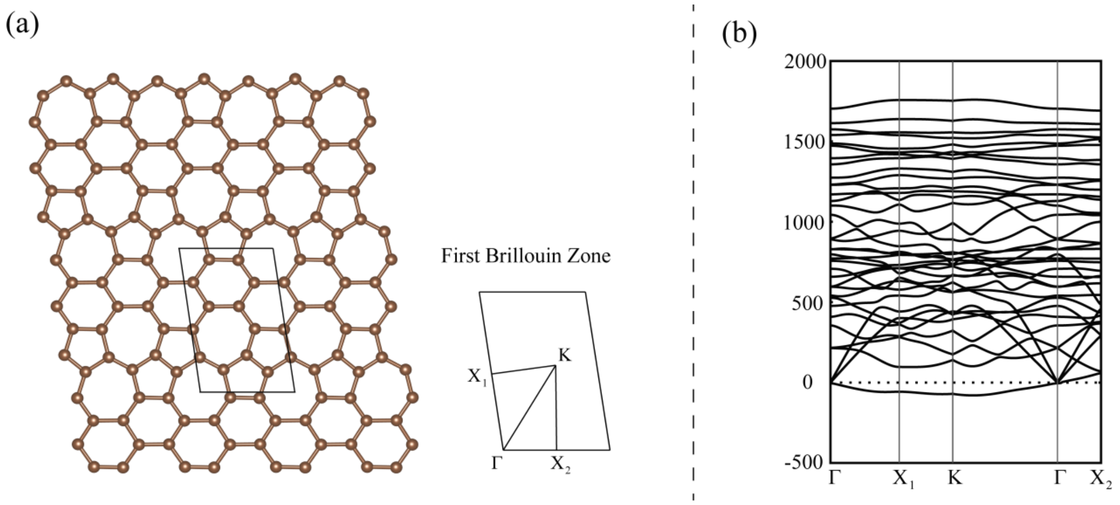

Fig. S2. (a) Two-dimensional crystal structure of HHPG-Z and its first Brillouin Zone. (b) Phonon dispersion along the typical directions.

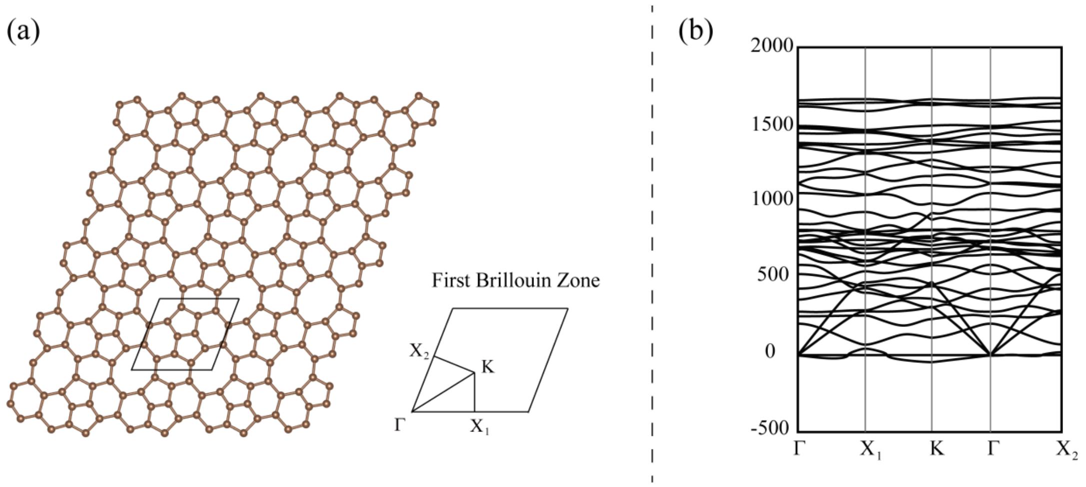

Fig. S3. (a) Two-dimensional crystal structure of OHPG and its first Brillouin Zone. (b) Phonon dispersion along the typical directions.
